# The effects of heat stress on morphological properties and intracellular signaling of denervated and intact soleus muscles in rats

**DOI:** 10.14814/phy2.13350

**Published:** 2017-08-07

**Authors:** Takashi Ohira, Akira Higashibata, Masaya Seki, Yoichi Kurata, Yayoi Kimura, Hisashi Hirano, Yoichiro Kusakari, Susumu Minamisawa, Takashi Kudo, Satoru Takahashi, Yoshinobu Ohira, Satoshi Furukawa

**Affiliations:** ^1^ Division of Aerospace Medicine Department of Cell Physiology The Jikei University School of Medicine Tokyo Japan; ^2^ Space Biomedical Research Group Japan Aerospace Exploration Agency Tsukuba Ibaraki Japan; ^3^ Japanese Experiment Module Utilization Center Japan Aerospace Exploration Agency Tsukuba Ibaraki Japan; ^4^ Advanced Engineering Services Co. Ltd. Tsukuba Ibaraki Japan; ^5^ Advanced Medical Research Center Yokohama City University Yokohama Kanagawa Japan; ^6^ Department of Cell Physiology The Jikei University School of Medicine Tokyo Japan; ^7^ Laboratory Animal Resource Center Department of Anatomy and Embryology Faculty of Medicine University of Tsukuba Tsukuba Ibaraki Japan; ^8^ Graduate School of Health and Sports Science Doshisha University Kyoto Japan

**Keywords:** Atrophy, heat stress, hypertrophy, neural innervation, skeletal muscle

## Abstract

The effects of heat stress on the morphological properties and intracellular signaling of innervated and denervated soleus muscles were investigated. Heat stress was applied to rats by immersing their hindlimbs in a warm water bath (42°C, 30 min/day, every other day following unilateral denervation) under anesthesia. During 14 days of experimental period, heat stress for a total of seven times promoted growth‐related hypertrophy in sham‐operated muscles and attenuated atrophy in denervated muscles. In denervated muscles, the transcription of ubiquitin ligase, atrogin‐1/muscle atrophy F‐box (*Atrogin‐1*), and muscle RING‐finger protein‐1 (*MuRF‐1*), genes was upregulated and ubiquitination of proteins was also increased. Intermittent heat stress inhibited the upregulation of *Atrogin‐1*, but not *MuRF‐1* transcription. And the denervation‐caused reduction in phosphorylated protein kinase B (Akt), 70‐kDa heat‐shock protein (HSP70), and peroxisome proliferator‐activated receptor *γ* coactivator‐1*α* (PGC‐1*α*), which are negative regulators of *Atrogin‐1* and *MuRF‐1* transcription, was mitigated. In sham‐operated muscles, repeated application of heat stress did not affect *Atrogin‐1* and *MuRF‐1* transcription, but increased the level of phosphorylated Akt and HSP70, but not PGC‐1*α*. Furthermore, the phosphorylation of Akt and ribosomal protein S6, which is known to stimulate protein synthesis, was increased immediately after a single heat stress particularly in the sham‐operated muscles. The effect of a heat stress was suppressed in denervated muscles. These results indicated that the beneficial effects of heat stress on the morphological properties of muscles were brought regardless of innervation. However, the responses of intracellular signaling to heat stress were distinct between the innervated and denervated muscles.

## Introduction

Skeletal muscles are important tissues for locomotion and metabolic regulation of the body. Skeletal muscle mass and contractile properties are affected by alteration in the neuromuscular activity level. In particular, chronic mechanical unloading causes unfavorable adaptations in the structure and function of skeletal muscles. For example, bed rest and spaceflight for humans (Convertino 1990; Ohira et al. 2000; Fitts et al. 2010) and hindlimb suspension and spaceflight for animals (Desplanches et al. 1987; Ohira et al. 1992; Sandonà et al. 2012) were shown to cause muscle mass loss and a shift in fiber phenotype toward a faster type, particularly in antigravity muscles composed predominantly of slow‐twitch fibers, such as the soleus (Ohira et al. 1992; Fitts et al. 2010; Sandonà et al. 2012) and adductor longus muscles (Riley et al. 1996; Ohira et al. 2011). These changes cause a decline in force generation and resistance to muscle fatigability (Yamashita‐Goto et al. 2001; Trappe et al. 2008; Fitts et al. 2010). The prescription of exercise is widely accepted as an effective intervention to maintain and improve skeletal muscle mass and contractile properties (Convertino 1990; Trappe et al. 2009; Fitts et al. 2010). In addition, denervation of skeletal muscles has been shown to result in severe atrophy of muscle fibers (Ohira 1989; Siu and Always 2005; O'Leary et al. 2012). However, effective countermeasures for denervation‐caused muscle atrophy have not been identified.

Such exogenous stimuli as electrical stimulation and vibration were recently reported to affect the maintenance of skeletal muscle mass and contractile properties (Salanova et al. 2014; Su et al. 2016). Heat is another notable stimulus that can be utilized as a countermeasure for the inconvenient skeletal muscle adaptation to chronic disuse in human and animals. An increase in muscle temperature to 38–42°C was shown to stimulate protein synthesis and result in skeletal muscle hypertrophy (Goto et al. 2011; Kakigi et al. 2011; Naito et al. 2012; Ohno et al. 2012; Yoshihara et al. 2013). Yoshihara et al. (2013) showed that phosphorylation of the protein kinase B (Akt) and 70‐kDa ribosomal protein S6 kinase (p70S6K) was significantly increased in rat soleus and plantaris muscles immediately following hindlimb immersion into warm water (41°C) for 30 min under anesthesia with *i.p*. injection of sodium pentobarbital. Akt and p70S6K are components of the Akt‐mammalian target of the rapamycin (mTOR) signaling cascade, which is an important pathway that causes skeletal muscle hypertrophy (Bodine et al. 2001b; Schiaffino et al. 2013).

Furthermore, heat stress has been shown to alleviate muscle atrophy caused by hindlimb unloading (Naito et al. 2000) or immobilization (Selsby and Dodd 2005; Sugiura et al. 2010). Naito et al. (2000) demonstrated that exposure to 41.0 ± 0.1°C for 60 min in a heat chamber suppressed protein loss in rat soleus muscles after 8 days of hindlimb suspension. Selsby and Dodd (2005) reported that intermittent heat stress under anesthesia using isoflurane while maintaining a core temperature of 41–45°C for 30 min using a thermal blanket was able to inhibit immobilization‐caused atrophy of the soleus muscles in rats. These studies indicated that heat stress may be an effective new treatment for skeletal muscles to promote hypertrophy and/or prevent atrophy. However, the mechanisms behind these observations and the effectiveness of heat stress treatment for denervation‐caused muscle atrophy have not been fully understood. Therefore, the effects of intermittent heat stress application on growth‐related hypertrophy and denervation‐caused atrophy of the soleus muscle and muscle fibers in rats were investigated. In addition, the effect of a single heat stress on the Akt‐mTOR cascade in sham‐operated and denervated soleus muscles was also determined in this study.

## Materials and Methods

### Experimental animals

All experimental procedures were conducted in accordance with the Guide for the Care and Use of Laboratory Animals of the Japanese Physiological Society. This study was approved by the Committee on Animal Care and Use at the Japan Aerospace Exploration Agency. Male Wistar Hannover rats (10 weeks old; Nihon CLEA, Tokyo, Japan) were utilized in the study. Each rat was housed in a plastic cage (27.6 (W) × 44.5 (L) × 20.4 (H) cm) in a temperature‐ and humidity‐controlled animal room (at approx. 24°C and 55%, respectively) with a 12:12 h light‐dark cycle. The rats had free access to a solid diet (CE‐2; Nihon CLEA, Tokyo, Japan) and water. The amount of food consumed by each rat was measured daily. All anesthesia procedures as indicated in the following experimental design were performed by isoflurane inhalation.

#### Experiment 1: The change in soleus muscle temperatures during heat stress

Five rats were used in the experiment. The left soleus muscle was exposed and a temperature probe (TOG212‐106a; Unique Medical, Tokyo, Japan) was inserted into the midbelly region of the muscle under anesthesia. The skin was sutured at the end of surgery. After 2 days of recovery, the soleus muscle temperatures were measured during anesthesia alone or heat stress under anesthesia.

#### Experiment 2: The effect of intermittent heat stress on the morphological properties and intracellular signaling of intact and denervated soleus muscles

Unilateral denervated rats were randomly divided into two groups: (1) heat stress application group (Heat, *n* = 8) and (2) control group without heat stress application (Cont, *n* = 8). And preexperimental control group (Pre‐exp, *n* = 8) was also prepared. Heat stress under anesthesia or only anesthesia was performed every other day (starting from the day following the unilateral denervation or sham operation). All rats, except for Pre‐exp group, were euthanized 14 days after the surgery. The rats in Pre‐exp group were euthanized before the surgery.

#### Experiment 3: The effect of a single heat stress on intracellular signaling of intact and denervated soleus muscles

Unilateral denervated rats were randomly divided into two groups: (1) heat stress application group (Heat, *n* = 10) and (2) control group without heat stress application (Cont, *n* = 10). Five rats in each group were euthanized immediately after heat stress under anesthesia or only anesthesia 1 day after the denervation surgery. And other rats were euthanized immediately after each treatment at 14 days after the denervation surgery.

### Unilateral denervation

The right sciatic nerve (approx. 5 mm) was cut to eliminate the influence of innervation, and the left nerve was sham operated under anesthesia. The skin was sutured at the end of surgery and the rats were allowed to recover in cages.

### Heat stress application

Heat stress was performed in the Heat group. The rats were subjected to hindlimb immersion using a warm water bath (42°C) for 30 min under anesthesia. During the procedure, the hindlimbs were covered with plastic to avoid direct contact of the skin with warm water. Rats in the Cont group received anesthesia only.

### Muscle preparation

The rats were euthanized immediately after each treatment under anesthesia and soleus muscles were removed bilaterally. The wet weight of the muscles was measured and the muscles were gently stretched to near optimal length in vivo, pinned on a cork, and frozen in isopentane cooled with liquid nitrogen. The samples were stored at −80°C before being analyzed.

### Analysis of muscle water content

Water content of the soleus muscle was calculated using the wet and dry muscle weights. Briefly, labeled aluminum foils were kept in an incubator (50°C) for a day to allow for complete dehydration. A section of each muscle sample was placed on the foil to measure its wet weight. The muscle samples were then incubated in a heat chamber set at 50°C. Muscle weights were assessed at days 1, 5, and 7 during incubation until stable dry weights were obtained. We confirmed that muscle water retention was completely removed based on a plateau of dry muscle weight. The weight at day 7 was recorded as the dry weight of each muscle section and the percentage of water content was calculated.

### Immunohistochemistry

The midbelly region of frozen soleus muscles was mounted perpendicularly on a cork using an optimum cutting temperature (OCT) compound (Sakura Finetek USA, CA) and immediately frozen in liquid nitrogen. Subsequently, 8‐*μ*m‐thick cross‐sections were sliced using a cryostat at −20°C and each section was placed on a glass slide. A standard immunohistochemistry staining procedure was performed in order to analyze the cross‐sectional areas (CSAs) of muscle fibers. Briefly, muscle sections were fixed in 4% paraformaldehyde for 15 min and preincubated in 0.1 mol/L phosphate‐buffered saline (PBS) containing 10% goat serum and 1% Triton X‐100 for 1 h at room temperature. A laminin rabbit polyclonal antibody (L9393; Sigma‐Aldrich, MO) was used as the primary antibody. The primary antibody was diluted 1:200 in 0.1 mol/L PBS containing 5% goat serum and 0.3% Triton X‐100, and then incubated with the muscle sections overnight at 4°C. Alexa Fluor 594‐conjugated goat antirabbit IgG antibody (R37117; Thermo Fisher Scientific, MA) was used as a secondary antibody. The secondary antibody was diluted 1:400 in 0.1 mol/L PBS containing 5% goat serum and 0.1% Triton X‐100, and then incubated with the muscle sections for 4 h at room temperature. Thereafter, muscle sections were mounted with 50% glycerol in 0.1 mol/L PBS and evaluated using a fluorescence microscope (BX53; OLYMPUS, Tokyo, Japan). Fiber CSAs were analyzed using Image‐Pro Analyzer (Version 7.0.1; Nippon Roper, Tokyo, Japan) image processing software. At least 600 fibers were analyzed for each muscle sample.

### Immunoblot analysis

Total protein was extracted from the proximal region of the frozen muscle by homogenization in a sample buffer containing 1% Triton X‐100, 50 mmol/L Tris‐HCL (pH 7.4), 4 mmol/L EGTA, 10 mmol/L EDTA, 10 mmol/L NaF, 2 mmol/L Na_3_VO_4_, 100 *μ*mol/L MG132, a complete protease inhibitor cocktail (Roche, Basel, Switzerland), and Phos STOP (Roche). Protein concentration was determined using the 2‐D Quant Kit (GE Healthcare Life Sciences, MA). The extracted protein (4 *μ*g/*μ*L) was mixed with 2 ×  sample buffer solution with 2‐mercaptoethanol for SDS‐PAGE (Nacalai Tesque, Kyoto, Japan) and heated at 60°C for 10 min. Equal amounts of protein (20 *μ*g) were separated on an SDS‐polyacrylamide gel (Mini‐PROTEAN TGX Gel, 4–20%; Bio‐Rad, CA) and transferred onto a polyvinylidene difluoride membrane using a Trans‐Blot Turbo Blotting System (Bio‐Rad). MagicMark XP Western Protein Standard (LC5602; Thermo Fisher Scientific) was used to estimate protein molecular weight (20–220 kDa).

Membranes were blocked with 5% skim milk diluted in Tris‐buffered saline containing 0.05% Tween 20 and then incubated with primary antibodies diluted at 1:1000–2000 using the Can Get Signal Immunoreaction Enhancer Solution (solution 1; TOYOBO, Osaka, Japan) overnight at 4°C. Membranes were then incubated with secondary antibodies (7076S or 7074S; Cell signaling Technology (CST) Japan, Tokyo, Japan) diluted at 1:2000 using the Can Get Signal Immunoreaction Enhancer Solution (solution 2; TOYOBO) for 1 h at room temperature. Signals were detected using the Luminata Forte Western HRP Substrate (Millipore, MA) and analyzed using a Chemiluminescence Imaging System (Ez‐Capture MG; ATTO, Tokyo, Japan). The phosphorylation levels of target proteins were evaluated using the same membrane. After signal detection, antibodies for phosphorylated proteins were stripped by washing with stripping buffer containing 62.5 mmol/L Tris (pH 6.8), 100 mmol/L 2‐mercaptoethanol, and 2% SDS.

#### Antibodies

The following primary antibodies were used for immunoblot analysis: Phospho‐Akt (Ser473) (9271S; CST Japan); Akt (9272S; CST Japan); Phospho‐Akt1 (Ser473) (9018S; CST Japan); Akt1 (75692S; CST Japan); Phospho‐Akt2 (Ser474) (8599S; CST Japan); Akt2 (3063S; CST Japan); Phospho‐Ribosomal Protein S6 (Ser235/236) (2211S; CST Japan); Ribosomal Protein S6 Antibody (2317; CST Japan); HSP70/HSP72 (ADI‐SPA‐810; Enzo Life Sciences, Tokyo, Japan); PGC‐1ɑ (ST1202; EMD Millipore, Darmstadt, Germany); Phospho‐AMPKɑ (Thr172) (Cell signaling, 2535S; CST Japan); AMPKɑ (2532S; CST Japan); GAPDH (2251‐1; Epitomics, CA); and Ubiquitin (3936S; CST Japan).

### Real‐time polymerase chain reaction (RT‐PCR)

Total RNA in the remaining portion of the soleus muscle sample was extracted using a reagent containing phenol and guanidine thiocyanate (ISOGEN; Nippon Gene, Toyama, Japan) as per the manufacturer's instructions. Total RNA was treated with RNase‐free DNase (Qiagen, Hilden, Germany) and 1 *μ*g of DNase‐treated total RNA was used for each cDNA synthesis reaction using the PrimeScript RT Master Mix (Takara Bio, Kyoto, Japan) as per the manufacturer's instructions. Polymerase chain reaction (PCR) was conducted with SYBR Green I (SYBR Premix Ex Taq; Takara Bio) using Light Cycler 96 (Roche, Basel, Switzerland). PCR amplification was performed for 45 cycles under conditions of 95°C for 30 sec, 57°C for 30 sec, and 72°C for 20 sec, in order to assess the atrogin‐1/muscle atrophy F‐box (*Atrogin‐1*) and muscle RING‐finger protein‐1 (*MuRF‐1*) gene expressions. TATA box‐binding protein (*Tbp*) was used as an internal control for RT‐PCR analysis (Nakao et al. 2015).

#### Primer sequences

The following primers were used for RT‐PCR analysis: *Atrogin‐1*: forward, 5′‐GGAAGCTTTCAACAGACTGGA‐3′ and reverse, 5′‐CTCAGGGATGTGAGCTGTGA‐3′; *MuRF‐1*: forward, 5′‐AGAAGAGTGAGCTGCTGCAG‐3′ and reverse, 5′‐TGGCTGTTTCCACAAGCTTG‐3′; *Tbp*: forward, 5′‐TGCCATGACTCCTGGAATTCC‐3′ and reverse, 5′‐TGCTGCTGCTGTCTTTGTTG‐3′.

### Statistical analysis

All data, except for the histograms of fiber CSA, were presented as the mean ± standard deviation (SD). Student's unpaired t‐test or two‐way ANOVA was performed using GraphPad Prism (Version 7.02; GraphPad Software, CA). When an interaction (Heat stress × Denervation) was observed, Tukey's multiple comparisons test was also performed. *P* values of < 0.05 were considered statistically significant.

## Results

### Experiment 1: The change in soleus muscle temperatures during heat stress

**Figure 1:**
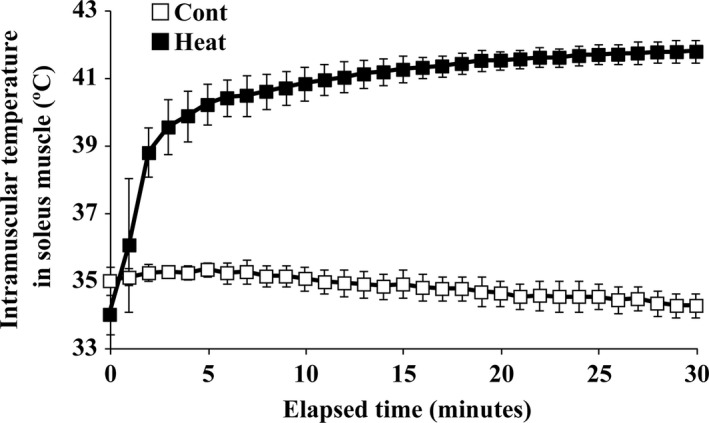
Time‐dependent changes in intramuscular temperature in soleus during heat stress under anesthesia (Heat) or anesthesia only (Cont). Hindlimbs of rats in the Heat group were immersed in a warm water bath (42°C) for 30 min during heat stress, while Cont group rats received anesthesia only. Means ± SD (*n* = 5 per group).

The time‐dependent changes in soleus muscle temperature were determined during heat stress under anesthesia or anesthesia alone. Soleus muscle temperature before the treatment was 34 ± 0.6°C, and increased to 41.8 ± 0.3°C within 30 min of heat stress application (Fig. 1). In contrast, the muscle temperature was gradually decreased from 35 ± 0.2°C to 34.3 ± 0.4°C during 30 min of anesthesia alone.

### Experiment 2: The effect of intermittent heat stress on the morphological properties and intracellular signaling of intact and denervated soleus muscles

#### Food consumption and body weight

The amount of daily food intake in the Heat (19.7 ± 1.7 g) and Cont (19.2 ± 1.2 g) groups was comparable during the experimental period. The mean body weight in Heat and Cont groups increased from 282 ± 14 g to 303 ± 15 g and from 281 ± 9 g to 306 ± 15 g, respectively, after 14 days. However, no significant difference in weight was observed between the Heat and Cont groups. Furthermore, the initial body weight of both groups was not statistically different with body weight of the Pre‐exp group (277 ± 5 g).

### Soleus muscle response to heat stress

#### Wet weight and water content

**Figure 2:**
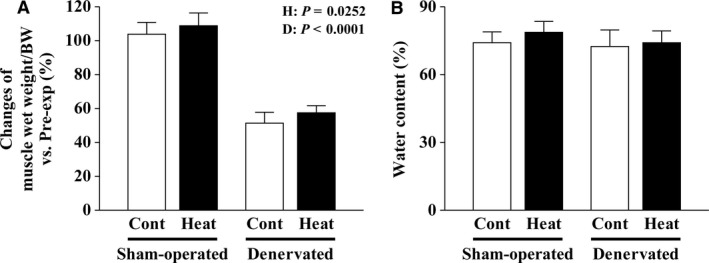
Effects of intermittent heat stress and/or 14 days of denervation on the percent changes in muscle wet weight/body weight (BW) versus the preexperimental control (Pre‐exp, A) and water content (B) in the sham‐operated and denervated soleus muscles. Cont: nonheated and Heat: heat stress applied groups. Mean ± SD (*n* = 8 per group). H and D: main effect of heat stress and denervation, respectively.

The growth‐related increase in soleus muscle weight was observed on the sham‐operated side (Fig. 2A). In contrast, the weight of denervated muscles was significantly decreased after 14 days (*P *<* *0.0001, Fig. 2A). Repeated application of heat stress augmented muscle hypertrophy and mitigated muscle atrophy (*P = *0.0252). The relative wet weight of sham‐operated and denervated soleus muscles to body weight in the Heat group was 5% and 12% higher, respectively, than each in the Cont group.

The mean water content in the sham‐operated and denervated soleus muscles was 74.0% and 72.4%, respectively, for the Cont group (Fig. 2B), but the difference was not statistically significant. In the Heat group, these values were 78.6% and 73.8%, respectively, although the difference was also not statistically significant (Fig. 2B).

#### Fiber CSAs

**Figure 3:**
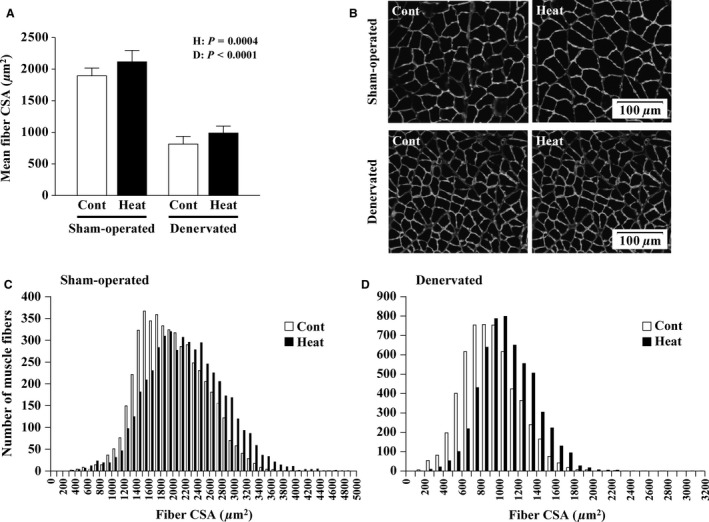
Effects of intermittent heat stress and/or 14 days of denervation on the morphological properties of soleus muscle fibers. Mean (± SD,* n* = 8 per group) fiber cross‐sectional areas (CSAs) are shown in A. Representative images of cross‐sections in sham‐operated and denervated muscles are shown in B. Muscle sections were stained for laminin to analyze fiber CSAs. The frequency distribution of fibers with different CSAs in sham‐operated (C) and denervated (D) muscles are also shown. At least 600 fibers in each muscle were analyzed. H and D: main effect of heat stress and denervation, respectively. See Figure 2 for other abbreviations.

The mean fiber CSAs in the sham‐operated and denervated muscles of the Heat group were 12% and 22% greater, respectively, than those of the Cont group (*P = *0.0004, Fig. 3A, B). The frequency distribution of fibers with different CSAs was shown in the histograms (Fig. 3C, D). There were clearly many thick muscle fibers in the Heat group.

#### Protein synthesis mechanism

**Figure 4:**
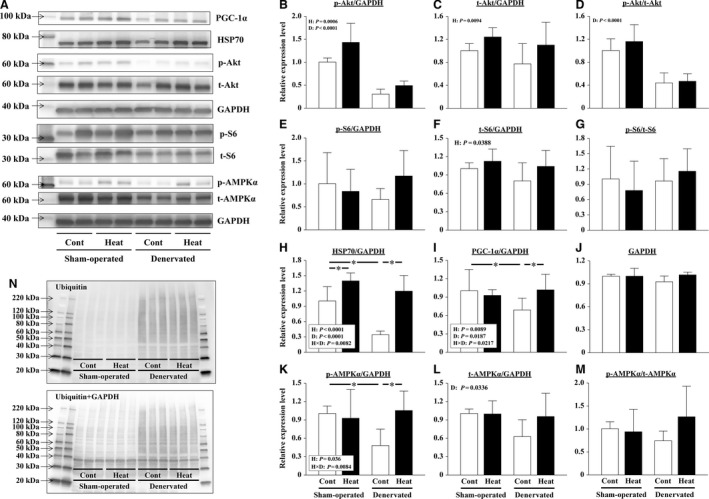
Effects of intermittent heat stress and/or 14 days of denervation on the protein expression levels in sham‐operated and denervated soleus muscles. The phosphorylated (p) and total (t) levels of protein kinase B (Akt), ribosomal protein S6 (S6), and AMP‐activated protein kinase *α* (AMPK *α*); the expression level of 70‐kDa heat‐shock protein (HSP70), peroxisome proliferator‐activated receptor‐*γ* coactivator‐1*α* (PGC‐1*α*), ubiquitinated proteins, and glyceraldehyde 3‐phosphate dehydrogenase (GAPDH) were evaluated by immune blotting. Representative results of immunoblotting are shown in A and N. The expression level of each protein was calculated after normalization using the expression levels of GAPDH (B, C, E, F, H, I, K, L). The phosphorylation rate of the protein was calculated by dividing the expression level of phosphorylated protein by the expression level of total protein (D, G, M). The relative values pertaining to the sham‐operated Cont muscles are also shown. Means ± SD (*n* = 8 per group). H and D: main effect of heat stress and denervation, respectively; H×D: interaction of effects of heat stress and denervation; *: the result of Tukey's multiple comparisons test was *P *<* *0.05. See Figure 2 for other abbreviations.

Activation of the Akt‐mTOR cascade in the muscles was evaluated. The level of phosphorylated Akt/GAPDH was significantly lower in denervated soleus muscles than that in sham‐operated muscles (*P *<* *0.0001, Fig. 4A, B). On the other hand, 14 days of denervation did not affect the level of total Akt/GAPDH (Fig. 4A, C). Therefore, Akt phosphorylation (phosphorylated/total Akt) in denervated muscles was significantly lower than that observed in the sham‐operated muscles (*P *<* *0.0001, Fig. 4A, D). Intermittent heat stress increased both levels of phosphorylated (*P = *0.0006) and total Akt (*P = *0.0094)/GAPDH in sham‐operated and denervated muscles (Fig. 4A–C). Neither intermittent heat stress nor denervation affected the expression level of GAPDH (Fig. 4A, J).

Both levels of phosphorylated and total ribosomal protein S6/GAPDH in soleus muscles were not influenced by 14 days of denervation (Fig. 4A, E, F, G). The total ribosomal protein S6/GAPDH level was significantly increased by intermittent heat stress in sham‐operated and denervated muscles (*P = *0.0388, Fig. 4A, F). However, the phosphorylation level of ribosomal protein S6 was not affected by heat stress (Fig. 4A, G).

#### Cytoprotection mechanism

The levels of HSP70/GAPDH (*P *<* *0.0001) and PGC‐1*α*/GAPDH (*P = *0.0073) were significantly reduced in the soleus muscles after 14 days of denervation (Fig. 4A, H, I). Repeated heat stress completely suppressed these reductions in HSP70/GAPDH (*P* < 0.0001) and PGC‐1*α*/GAPDH (*P = *0.0057) (Fig. 4A, H, I). At the same time, the heat stress significantly increased HSP70/GAPDH in sham‐operated soleus muscles (*P = *0.01), but did not affect the PGC‐1*α* level (Fig. 4A, H, I).

Furthermore, the phosphorylation level of AMPK*α* was not influenced by intermittent heat stress in both sham‐operated and denervated soleus muscles (Fig. 4A, M). However, the level of both total and phosphorylated AMPK*α*/GAPDH appeared to be reduced in response to denervation (Fig. 4A, K, L). In particular, the level of phosphorylated AMPK*α*/GAPDH was significantly less in denervated Cont muscles than in the sham‐operated Cont muscles (*P = *0.0159, Fig. 4A, K). And intermittent heat stress was able to prevent the denervation‐caused reduction (*P = *0.0069, Fig. 4A, K).

#### Protein degradation mechanism

**Figure 5:**
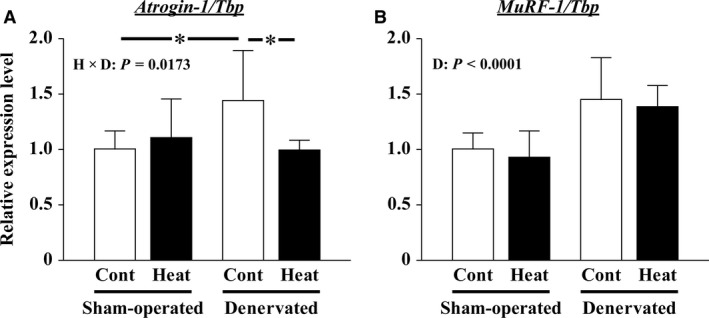
Effects of intermittent heat stress and/or 14 days of denervation on the transcription of atrogin‐1/muscle atrophy F‐box (*Atrogin‐1*, A) and muscle RING‐finger protein‐1 (*MuRF‐1*, B) genes in soleus muscles were evaluated by real‐time polymerase chain reaction (RT‐PCR). The expression levels of *Atrogin‐1* and *MuRF‐1 mRNA* were obtained by calculation after normalization using the expression level of *TATA box‐binding protein* (*Tbp*). The relative expression levels pertaining to the sham‐operated Cont muscles are also shown. Mean ± SD (*n* = 8 per group). D: main effect of denervation, respectively; H×D: interaction of effects of heat stress and denervation; *: the result of Tukey's multiple comparisons test was *P *<* *0.05. See Figure 2 for other abbreviations.

Fourteen days of denervation increased the expression of *Atrogin‐1* and *MuRF‐1,* muscle‐specific E3 ubiquitin ligases, genes (Fig. 5A, B). However, the transcription of *Atrogin‐1* in denervated muscles was significantly suppressed to the level in the sham‐operated Cont muscle by heat stress (*P = *0.0312, Fig. 5A). The increase in *MuRF‐1* transcription was not affected by intermittent heat stress (Fig. 5B). In sham‐operated muscles, the expression of *Atrogin‐1* and *MuRF‐1* genes was not affected by heat stress (Fig. 5A, B).

The abundance of total ubiquitinated proteins was determined by immunoblot analysis. The ubiquitination of proteins increased markedly after 14 days of denervation (Fig. 4N). However, the ubiquitination in denervated muscles was slightly suppressed by the repeated application of heat stress. Heat stress did not affect the protein ubiquitination in sham‐operated soleus muscles.

#### Experiment 3: The effect of a single heat stress on intracellular signaling of intact and denervated soleus muscles

Yoshihara et al. (2013) has reported that heat stress activates the Akt‐mTOR cascade in soleus muscles. However, as shown above, it was not confirmed in our “Experiment 2.” Thus, “Experiment 3” was performed to determine the responses of the Akt‐mTOR cascade in soleus muscles immediately after a single heat stress.

### Responses to heat stress in soleus muscles a day after sham operation or denervation

**Figure 6:**
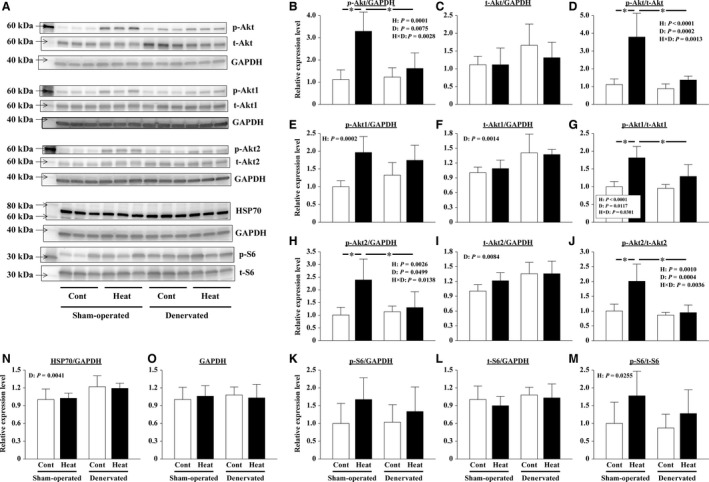
Effects of a single heat stress and/or 1 day of denervation on the protein expression levels in sham‐operated and denervated soleus muscles. The phosphorylated (p) and total (t) levels of Akt, Akt1, Akt2, and S6; the expression level of HSP70 and GAPDH was evaluated by immune blotting. Representative results of immunoblotting are shown in A. The expression level of each protein was calculated after normalization using the expression levels of GAPDH (B, C, E, F, H, I, K, L, N). The phosphorylation rate of the protein was calculated by dividing the expression level of phosphorylated protein by the expression level of total protein (D, G, J, M). The relative values pertaining to the sham‐operated Cont muscles are also shown. Means ± SD (*n* = 5 per group). H and D: main effect of heat stress and denervation, respectively; H×D: interaction of effects of heat stress and denervation; *: the result of Tukey's multiple comparisons test was *P *<* *0.05. See Figures 2 and 4 for other abbreviations.

Immediately after a single heat stress, the level of phosphorylated (not total) Akt/GAPDH was drastically increased in sham‐operated soleus muscles (*P *<* *0.0001, Fig. 6A–C). Therefore, the level of Akt phosphorylation was significantly increased in the sham‐operated muscles (*P *<* *0.0001, Fig. 6D). However, the effects of heat stress were significantly reduced in denervated soleus muscles (Fig. 6A–D).

Similar responses as above were observed for both different isoforms (Akt1 and Akt2) of the Akt family. Heat stress increased the levels of phosphorylated Akt1/GAPDH (*P = *0.0002) and Akt2/GAPDH (*P = *0.0026) in both sham‐operated and denervated soleus muscles (Fig. 6A, E, H). However, the increasing level of phosphorylated Akt2/GAPDH, but not Akt1/GAPDH, was lightened in denervated muscles. And denervation also increased the levels of total Akt1/GAPDH (*P = *0.0014, Fig. 6A, F) and Akt2/GAPDH (*P = *0.0084, Fig. 6A, I). As a result, the phosphorylation levels of Akt1 and Akt2 were significantly increased by heat stress only in the sham‐operated muscles (Fig. 6G, J). Neither a single heat stress nor denervation affected the expression level of GAPDH (Fig. 6A, O).

The levels of phosphorylated ribosomal protein S6/GAPDH tended to be increased by heat stress in both sham‐operated and denervated soleus muscles (*P = *0.0626, Fig. 6A, K). Conversely, the levels of total ribosomal protein S6/GAPDH were not affected by heat stress and denervation (Fig. 6A, L). Therefore, the heat stress‐brought increasing phosphorylation levels of ribosomal protein S6 were observed in both sham‐operated and denervated muscles (*P = *0.0255, Fig. 6M).

The levels of HSP70/GAPDH were slightly but significantly increased a day after denervation (*P = *0.0041, Fig. 6A, N).

### Responses to heat stress in soleus muscles 14 days after sham operation or denervation

**Figure 7:**
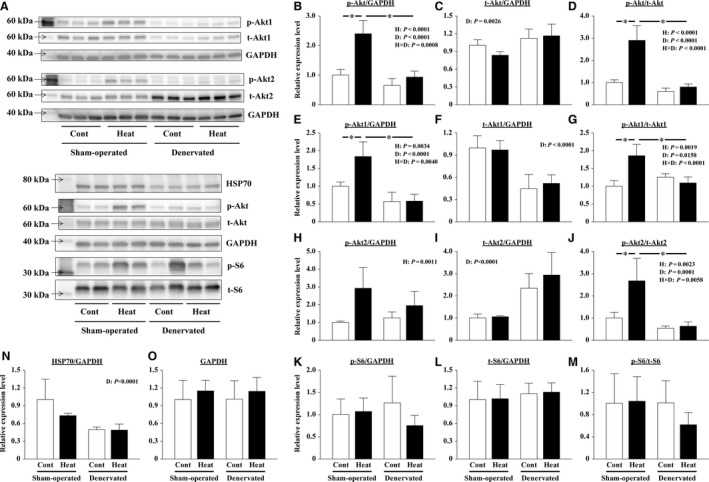
Effects of a single heat stress and/or 14 days of denervation on the protein expression levels in sham‐operated and denervated soleus muscles. The phosphorylated (p) and total (t) levels of Akt, Akt1, Akt2, and S6; the expression levels of HSP70 and GAPDH were evaluated by immune blotting. Representative results of immunoblotting are shown in A. The expression level of each protein was calculated after normalization using the expression levels of GAPDH (B, C, E, F, H, I, K, L, N). The phosphorylation rate of the protein was calculated by dividing the expression level of phosphorylated protein by the expression level of total protein (D, G, J, M). The relative values pertaining to the sham‐operated Cont muscles are also shown. Means ± SD (*n* = 5 per group). H and D: main effect of heat stress and denervation, respectively; H×D: interaction of effects of heat stress and denervation; *: the result of Tukey's multiple comparisons test was *P *<* *0.05. See Figure 2 and 4 for other abbreviations.

Responses of Akt to a single heat stress in soleus muscles 14 days after the sham operation or denervation was similar to those in the muscles a day after the surgery. Immediately after a single heat stress, the level of phosphorylated (not total) Akt/GAPDH was markedly increased in sham‐operated soleus muscles (*P *<* *0.0001, Fig. 7A–C). Therefore, the levels of phosphorylated/total Akt were significantly increased in the sham‐operated muscles (*P *<* *0.0001, Fig. 7D). However, the effects of heat stress were significantly reduced in denervated soleus muscles (Fig. 7A, B, D).

Heat stress increases the levels of phosphorylated Akt1/GAPDH (*P = *0.0034) and Akt2/GAPDH (*P = *0.0011) in both sham‐operated and denervated soleus muscles (Fig. 7A, E, H). The increasing level of phosphorylated Akt1/GAPDH, but not Akt2/GAPDH, was lightened by denervation. And 14 days of denervation, unlike a day after, decreased the levels of total Akt1/GAPDH (*P *<* *0.0001, Fig. 6F and Fig. 7F) and increased the total Akt2/GAPDH levels (*P *<* *0.0001, Fig. 7I). However, the phosphorylation levels of Akt1 and Akt2 were significantly increased by heat stress only in the sham‐operated muscles (Fig. 7G, J). Neither a single heat stress nor 14 days of denervation affected the expression level of GAPDH (Fig. 7A and O).

At this time, the levels of phosphorylated and total ribosomal protein S6/GAPDH were not affected by heat stress and denervation (Fig. 7A, K, L, M). Meanwhile, the levels of HSP70/GAPDH were significantly decreased by 14 days of denervation (*P* < 0.0001, Fig. 7A, N). However, no effect of single heat stress application on HSP70 content was observed.

## Discussion

The intermittent heat stress could promote muscle fiber hypertrophy and prevent atrophy of the soleus muscle in rats. Muscle mass is known to be regulated by the net balance between protein synthesis and degradation (Sandri 2008; Schiaffino et al. 2013). Therefore, the application of external heat may affect intracellular signaling that stimulates protein synthesis and inhibits protein degradation in the skeletal muscles. However, the responses of intact or denervated soleus muscles to heat stress were different. Our data indicated that innervation might be important for the activation of Akt by heat stress.

### Heat stress in promoting soleus muscle hypertrophy

Elevated wet weight and fiber CSA in the soleus muscle were observed following repeated application of heat stress (every other day) during the 14‐day experimental period (Fig. 2A and Fig. 3). At this time, an excessive increase in water content was not confirmed (Fig. 2B). These results were in agreement with previous reports (Goto et al. 2011; Naito et al. 2012; Ohno et al. 2012).

#### Effect of heat stress on protein synthesis in sham‐operated soleus muscles

The Akt‐mTOR cascade plays a crucial role in the regulation of protein synthesis (Bodine et al. 2001b; Sandri 2008; Schiaffino et al. 2013). The activation of this signaling pathway results in increased muscle mass. Kakigi et al. (2011) reported that heat stress application using a microwave therapy unit enhanced the activation of Akt‐mTOR signaling in human skeletal muscles after resistance exercise. Yoshihara et al. (2013) also reported that the phosphorylation of Akt‐mTOR signaling components Akt and p70S6K was significantly increased in soleus and plantaris muscles immediately after warm water immersion (41°C for 30 min). Consistent with previous studies, the phosphorylation of Akt increased significantly in sham‐operated soleus muscles immediately after a single heat stress in our “Experiment 3” (Fig. 6D and Fig. 7D). At this time, Akt1 and Akt2, the different isoforms of the Akt family, responded similarly to heat stress (Fig. 6G, J and Fig. 7G, J). Akt1 and Akt2 are highly expressed in skeletal muscle and appear to have distinct functions (Yang et al. 2004; Sandri 2008; Rotwein and Wilson 2009; Zheng and Cartee 2016). Akt1 is essential for skeletal muscle growth, whereas Akt2 is important for insulin‐stimulated glucose metabolism (Yang et al. 2004; Sandri 2008; Zheng and Cartee 2016). Therefore, our results suggest that heat stress would stimulate not only hypertrophy of muscle fibers but also glucose metabolism in intact skeletal muscles. Nevertheless, our study did not obtain consistent and clear results for ribosomal protein S6, which is downstream of Akt (Fig. 6M and Fig. 7M). Each activation timing of Akt and S6 which responded to heat stress might be different, although the detailed reasons are unknown.

On the other hand, elevated phosphorylation of Akt and ribosomal protein S6 was not observed in the soleus muscles sampled 24 h after the final heat stress application in “Experiment 2” (Fig. 4D). Our results indicated that heat stress can stimulate the Akt‐mTOR signaling cascade and promote protein synthesis in the innervated soleus muscles; however, the duration of the effect might be limited to within 24 h after heat application.

In “Experiment 2”, the intermittent application of heat stress significantly increased HSP70/GAPDH in sham‐operated soleus muscles (*P = *0.01, Fig. 4A, H). Locke and Celotti reported that HSP70 content was elevated in both soleus and extensor digitorum longus muscles after 24 h from a heat stress (42°C, 15 min) (Loche and Celotti 2014). Their data also showed that heat stress further increased the basically high expression level of HSP70 in soleus muscles. Gwag et al. (2013) summarized a potential signaling network in which HSP70 may play an important role in protein synthesis by activating Akt‐mTOR signaling. Therefore, it was considered the possibility that the increase in HSP 70 contributes to the activation of Akt by heat stress. However, in this study, the HSP70 protein level was not positively correlated with Akt phosphorylation as described here. HSP70 was significantly increased 1 day after the final application of intermittent heat stress in the sham‐operated muscles (Fig. 4H); however, the phosphorylation level of Akt was comparable in the Heat and Cont groups in “Experiment 2” (Fig. 4D). Furthermore, the level of phosphorylated Akt, but not HSP70, was significantly increased immediately after heat stress in “Experiment 3” (Fig. 6B, N and Fig. 7B, N). These results indicated that the expression of HSP70 and the activation of Akt‐mTOR signaling are not always positively correlated, especially in heat‐stressed soleus muscles.

#### Effect of heat stress on protein degradation in sham‐operated soleus muscles

Ubiquitin–proteasome‐dependent protein degradation plays a key role in muscle atrophy. In this proteolytic system, three enzymes (E1‐3) that contribute to the polyubiquitination of proteins and polyubiquitinated proteins are degraded by proteasome (Schiaffino et al. 2013; Bodine and Baehr 2014). The transcription of muscle‐specific E3 ubiquitin ligases, such as *Atrogin‐1* and *MuRF‐1*, has been used as a marker to estimate the level of protein degradation (Bodine et al. 2001a; Gomes et al. 2001). Furthermore, a link between protein degradation and synthesis pathways has been reported (Schiaffino et al. 2013). The upregulation of ubiquitin ligase gene transcription is typically suppressed by HSP70 overexpression and Akt activation via suppressing the function of the Forkhead box O (FoxO) transcription factor (Senf et al. 2010; Schiaffino et al. 2013). However, in this study, the expression of both *Atrogin‐1* and *MuRF‐1* genes did not differ between the Heat and Cont groups in sham‐operated muscles, despite the levels of HSP70 and phosphorylated Akt were significantly greater in the Heat group (Fig. 4B, H). The findings suggested that protein degradation was not affected by heat stress in intact soleus muscles.

### Heat stress attenuation of denervation‐caused soleus muscle atrophy

The denervation‐caused atrophy of soleus muscles was partially, but significantly, prevented by the application of intermittent heat stress (Figs. 2 and 3). Naito et al. (2000) also demonstrated that prior exposure to a thermic environment (41°C for 60 min) using a heat chamber suppressed rat soleus muscle atrophy caused by 8‐day hindlimb suspension. However, another study demonstrated that heat stress application using the same procedure was unable to prevent soleus muscle atrophy caused by 14‐day hindlimb suspension (Takeda et al. 2009). These results suggested that the repeated application of heat stress may be required to prevent skeletal muscle atrophy due to long‐term disuse. Recently, Tamura et al. (2015) reported that daily exposure to a heat environment in a chamber (40°C for 30 min) relieved the denervation‐caused gastrocnemius muscle atrophy in mice. The same result was confirmed in “Experiment 2” by using rat soleus muscles.

#### Effect of heat stress on protein synthesis in denervated soleus muscles

Heat stress increased phosphorylation of Akt in sham‐operated soleus muscles (Fig. 6D and Fig. 7D). And the increase was significantly suppressed by denervation. This phenomenon was similarly observed in the muscles a day after and 14 days after the denervation surgery. However, the total Akt1/GAPDH (*P* = 0.0014) and Akt2/GAPDH (*P* = 0.0084) levels increased significantly at 1 day after denervation (Fig. 6F and I). On the other hand, at 14 days after denervation, the levels of total Akt1/GAPDH (*P* < 0.0001) decreased and the total Akt2/GAPDH levels (*P* < 0.0001) increased (Fig. 7F, I). Similar results were also reported by Norrby et al. (2012) and MacDonald et al. (2014). Due to the high levels of total proteins, it is likely that the phosphorylation rates of Akt in denervated muscles were calculated low even immediately after heat stress. However, the heat stress‐caused increases in the phosphorylated proteins in denervated muscles are smaller than those in sham‐operated muscles (Fig. 6E, H and Fig. 7E, H). Therefore, we considered that phosphorylation of Akt by heat stress was suppressed by denervation.

The effect of heat stress on the phosphorylation of ribosomal protein S6 in denervated muscles has not been elucidated, as that in sham‐operated muscles. However, our results indicated that the peripheral nervous system plays an important role at least when heat stress activates Akt (Akt1 and Akt2). Therefore, we speculated that the activation of the Akt‐mTOR cascade may not be involved in the mitigation of muscle atrophy accompanying denervation by heat stress.

#### Effect of heat stress on protein degradation in denervated soleus muscles

The transcription of *Atrogin‐1* and *MuRF‐1* genes was upregulated by 14 days of denervation (Fig. 5). The result indicated that protein degradation was activated during the 14‐day denervation. However, the transcription of *Atrogin‐1*, but not *MuRF‐1*, was suppressed by intermittent application of heat stress (Fig. 5). In a previous study, *Atrogin‐1‐* and *MuRF‐1*‐deficient mice were shown to be resistant to muscle atrophy (Bodine et al. 2001a; Gomes et al. 2001; Cohen et al. 2012). In addition, the immunoblot analysis results of total ubiquitinated proteins also suggested that the ubiquitination in denervated muscles was slightly suppressed by the intermittent application of heat stress (Fig. 4N). Thus, heat stress was speculated to attenuate denervation‐caused soleus muscle atrophy, based on the partial suppression of protein degradation through the ubiquitin–proteasome system. Although we have not considered it in this study, the possibility that heat stress also suppresses other proteolytic systems, such as the calpain and/or autophagy system, has been reported in previous studies (Sugiura et al. 2010; Tamura et al. 2015; Yoshihara et al. 2015).

FoxO and nuclear factor *κ*B (NF‐*κ*B) are key transcription factors involved in the activation of *Atrogin‐1* and *MuRF‐1* gene promoters in skeletal muscle atrophy (Reed et al. 2011; Schiaffino et al. 2013; Bodine and Baehr 2014; Wu et al. 2014). Senf et al. (2010) revealed that FoxO3a can differentially regulate the activity of *Atrogin‐1* and *MuRF‐1* promoters. They demonstrated that HSP70 overexpression suppressed FoxO3a‐related transcription of *Atrogin‐1*, but not *MuRF‐1*, through Akt‐dependent and independent mechanisms. Furthermore, *MuRF1* transcription was reported to be controlled by NF‐*κ*B rather than by FoxO (Wu et al. 2014). And by using a plasmid‐mediated overexpression system, the rescue of disuse‐related decrease in HSP70 was shown to inhibit skeletal muscle atrophy (Senf et al. 2008, 2010; Dodd et al. 2009). These results suggested a crucial role of HSP70 in the maintenance of skeletal muscle mass. In this study, it is also likely that HSP70 contributed to the suppression of proteolysis by intermittent heat stress via the inhibition of *Atrogin‐1* transcription in denervated soleus muscles (Fig. 4H). However, the inhibition of muscle atrophy appeared to be minor, but statistically significant (Figs. 2 and 3), despite the complete blockage of any denervation‐related decrease in HSP70 (*P *<* *0.0001, Fig. 4H). Thus, our results also suggested the role of other factor(s) to mediate the beneficial effects of heat stress.

The disuse muscle atrophy was recently shown to be associated with mitochondrial loss and dysfunction (Siu and Always 2005; O'Leary et al. 2012; Powers et al. 2012; Talbert et al. 2013). PGC‐1*α* is a master regulator of mitochondrial biogenesis and function (Wu et al. 1999; Czubryt et al. 2003; Adhihetty et al. 2009). Baldelli et al. (2014) reported that PGC‐1*α* can buffer oxidative stress by maintaining the expression of antioxidant enzymes, and support myogenesis. PGC‐1*α* is also thought to be an important mediator of exercise‐brought cytoprotection. (Russell et al. 2003; Taylor et al. 2005; Ruas et al. 2012; Wiggs 2015). PGC‐1*α* in the muscle is decreased at an early stage of denervation or hindlimb unloading (Sandri et al. 2006; Sacheck et al. 2007; Kang and Ji 2013). Sandri et al. (2006) reported that denervation‐caused muscle atrophy can be mitigated by forced PGC‐1*α* expression via the suppression of FoxO3a activity and subsequent *Atrogen‐1* transcription. The volume of soleus and gastrocnemius muscles was preserved following 3‐ and 14‐day hindlimb unloading in transgenic mice that overexpressed PGC‐1*α* due to the suppression of protein degradation by autophagy and the ubiquitin–proteasome system (Cannavino et al. 2014, 2015). In this study, intermittent heat stress completely suppressed the denervation‐caused reduction in PGC‐1*α*/GAPDH (*P = *0.0057, Fig. 4A, I). The reduction in PGC‐1*α* was also slightly alleviated in the denervated gastrocnemius muscles of mice by a daily application of heat stress at 40°C for 30 min for 7 days (Tamura et al. 2015).

The activity of PGC‐1*α* is enhanced by its phosphorylation and deacetylation by AMPK*α* and SIRT1, respectively (Jäger et al. 2007; Schilling and Kelly 2011; Sanchez et al. 2012), and the activation of AMPK*α* is also required for the upregulation of a PGC‐1*α* level (Lee et al. 2006; Suwa et al. 2006; Jäger et al. 2007). In this study, the phosphorylation level of AMPK*α* in denervated soleus muscles was not influenced by intermittent heat stress (Fig. 4M). However, the reduction in the level of phosphorylated AMPK*α*/GAPDH by denervation was significantly suppressed by heat stress (Fig. 4A, K). Given the similar abundance of both PGC‐1*α* and phosphorylated AMPK*α*, the activity of PGC‐1*α* was expected to be enhanced in the denervated muscles of the Heat group as well as sham‐operated muscles of the Cont group. Furthermore, Xu et al. (2016) reported that PGC‐1*α* is crucial for the heat‐shock factor 1‐dependent induction of HSP70 in response to hyperthermia. Therefore, suppression of the decrease in PGC‐1*α* in denervated soleus muscles by intermittent heat stress may be related to suppression of the decrease in HSP70. As for the reason why, we considered that PGC‐1*α* can also contribute to the partial suppression of protein degradation.

## Conclusions

The intermittent application of heat stress promoted hypertrophy of intact soleus muscle fibers and attenuated denervation‐caused atrophy. Phosphorylation of Akt (Akt1 and Akt2) and ribosomal protein S6, which stimulates protein synthesis, was increased immediately after a single heat stress application in sham‐operated soleus muscles. The activation of the Akt‐mTOR cascade by heat stress was suppressed in the denervated muscles. Instead, repeated heat stress suppressed the reduction in negative regulators, including phosphorylated Akt, HSP70, and PGC‐1*α*; it also inhibited the upregulation of Atrogin‐1 (but not MuRF‐1) transcription in denervated muscles. And the ubiquitination of proteins was slightly suppressed. However, in sham‐operated muscles, heat stress did not affect the transcription of *Atrogin‐1* and *MuRF‐1*, despite the apparent increase in phosphorylated Akt and HSP70, but not PGC‐1*α*. These results indicated that the beneficial effects of heat stress on the morphological properties of the muscles were brought regardless of innervation. However, the effect of heat stress on intracellular signaling that regulates protein synthesis and degradation was different in innervated and denervated muscles. Together, these results suggested the potential use of heat stress as a new therapeutic intervention strategy for muscle wasting disorders associated with traumatic nerve injury or neuropathy.

## Conflict of Interest

The authors declare no conflict of interest.
